# Association Between Weight Gain From Young to Middle Adulthood and Metabolic Syndrome Across Different BMI Categories at Young Adulthood

**DOI:** 10.3389/fendo.2021.812104

**Published:** 2022-02-15

**Authors:** Xiuling Wang, Jiali Song, Yan Gao, Chaoqun Wu, Xingyi Zhang, Teng Li, Jianlan Cui, Lijuan Song, Wei Xu, Yang Yang, Haibo Zhang, Jiapeng Lu, Xi Li, Jiamin Liu, Xin Zheng

**Affiliations:** ^1^ National Clinical Research Center for Cardiovascular Diseases, National Health Commission (NHC) Key Laboratory of Clinical Research for Cardiovascular Medications, State Key Laboratory of Cardiovascular Disease, Fuwai Hospital, Chinese Academy of Medical Sciences and Peking Union Medical College, National Center for Cardiovascular Diseases, Beijing, China; ^2^ National Clinical Research Center of Cardiovascular Diseases, Coronary Artery Disease Center, Fuwai Hospital Chinese Academy of Medical Sciences, Shenzhen, China

**Keywords:** weight gain, young adulthood, middle adulthood, metabolic syndrome, BMI categories

## Abstract

**Objectives:**

We aimed to assess the dose–response association between weight gain from young to middle adulthood and odds of metabolic syndrome, across body mass index (BMI) categories at young adulthood.

**Methods:**

Based on a national population-based screening project, middle-aged (35–64 years) participants who recalled weight at age 25 years and received standardized measurements were included. Multivariable adjusted restricted cubic splines and logistic regression models were applied.

**Results:**

In total, 437,849 participants were included (62.1% women, 52.0 ± 7.6 years). Larger weight gains from young to middle adulthood were associated with higher odds of metabolic syndrome at middle adulthood, with odds of 2.01 (1.98–2.05), 1.93 (1.92–1.94), and 1.67 (1.64–1.7) per 5-kg weight gain across participants who were underweight, normal-weight, and overweight/obese at young adulthood, respectively. After further adjusting for current BMI, larger weight gains still correlated with higher odds of metabolic syndrome among underweight and normal-weight participants, while an inverted U-shaped association was observed in overweight/obese participants.

**Conclusions:**

Weight maintenance from young to middle adulthood could be effective to mitigate metabolic syndrome burden, especially among underweight and normal-weight people. Historical weight gain confers varied information about metabolic syndrome risk independent of attained BMI across BMI categories at young adulthood.

## Introduction

Metabolic syndrome, a cluster of metabolic risk factors, significantly contributes to the occurrence of cardiovascular disease (CVD) (1, 2). Recently, approximately 25% of the global population suffered from it (3). In China, the proportion of population with metabolic syndrome had reached 33.9% in 2010 (4). The metabolic disease burden is expected to increase as the prevalence of obesity, an important determinant for metabolic syndrome (5), continues to grow. People usually gain their body weight with increasing age, especially during the period from young to middle adulthood (6). Therefore, exploring the cumulative effects of weight gain from young to middle adulthood on the risk of metabolic syndrome is essential for comprehensively understanding the development of metabolic syndrome at middle adulthood.

Prior studies have shown that weight gain from young to middle adulthood increases metabolic syndrome risk (7–13). It is likely that body mass index (BMI) categories at young adulthood, partly reflecting genetic disposition (14), body composition, and health behaviors, may modify the association between weight gain from young to middle adulthood and metabolic syndrome risk. Yet, limited evidence exists in this research area. Few studies explored the effect modification of BMI categories at young adulthood, while those studies often had small sample sizes and did not consider the important confounder of weight change amount (7, 12). In addition, evidence with regard to underweight people is lacking. Moreover, a prior study found that adult weight gain exerted specific effects on the occurrence of metabolic syndrome independent of the current BMI. This also partly explains the heterogeneity in the cardiometabolic risk among the people with similar BMI (10). However, whether the independent effects of weight gain on the risk of metabolic syndrome interact with BMI categories at young adulthood has not been investigated. Extensively exploring the association between adult weight gain and metabolic syndrome across BMI categories at young adulthood could be critical to facilitate developing more detailed weight management strategy for prevention of metabolic syndrome and its associated unfavorable health outcomes.

Accordingly, using data from a large, nationwide population-based project, China Patient-Centered Evaluative Assessment of Cardiac Events Million Persons Project (PEACE MPP), we aimed to: 1) assess the dose–response association of weight gain from young to middle adulthood with odds of metabolic syndrome among all participants and across different BMI categories at young adulthood; and 2) investigate the independent association of weight gain from young to middle adulthood with metabolic syndrome across different BMI categories at young adulthood after accounting for the current BMI.

## Materials and Methods

### Study Design and Participants

The PEACE MPP is a government-funded public health program focusing on CVD risk throughout China. Details of the project have been described previously (15). In brief, from September 2015 to March 2020, the project sampled 286 sites (170 rural counties and 116 urban districts) in 31 provinces of mainland China. The typical case sampling design was applied to provide diversity in geographical distribution, economic development and population structure. Local residents aged 35–75 years were invited to the project by local staff *via* extensive publicity campaigns on television and in newspapers. Individuals enrolled for screening were asked to have measurements taken. Among the 3.6 million screened participants, 1.1 million (1,064,164) participants whose project ID number ended with 1, 3, 5, or 7 received an intensive in-person interview on individual body weight at the age of 25 and detailed information on lifestyle and medical history.

In this study, we included participants at middle age (35–64 years) who received intensive in-person interviews (n = 819,792). Participants who did not recall weight at age 25 years (n = 264,859), or who did not have measured weight or height during the screening visits (n = 767) were excluded, and participants with missing components of metabolic syndrome (blood pressure, blood glucose, blood lipids and waist circumference) (n = 12,861) or covariates (n = 16,701) were also excluded. Among participants with complete data (n = 524,604), we further excluded 85,662 participants who experienced weight loss during the period from age 25 years to middle age. In addition, participants who had self-reported cardiometabolic diseases including diabetes, hypertension, dyslipidemia, and cardiovascular diseases at age 25 years were also excluded (n = 1,093). A total of 437,849 participants were included in the study (**Figure S1**).

The central ethics committee at Fuwai Hospital approved this project. All enrolled participants provided written informed consent.

### Data Collection and Variable Definition

First, all measurements of participants at middle age were taken using standardized devices and procedures in the clinics. The blood pressure value was obtained by the means of two measurements on the right upper arm after 5 minutes of rest with a 1-minute interval (Omron HEM-7430, Omron Corporation, Japan). If there was a difference larger than 10 mmHg between two systolic blood pressure readings, a third measurement was taken and the mean value of the last two measurements was used. Trained technicians measured the height and weight of participants who were required to wear light clothes, no shoes and no cap. The degree of accuracy was to the nearest 0.1 kg and 0.1 cm separately for weight and height. Waist circumference was measured midway between the lower edge of the costal arch and the upper edge of the iliac crest and to the nearest 0.1 cm. Participants received lipid blood test and glucose blood test by unified devices using whole blood samples. TG and HDL-C were measured by a rapid lipid analyzer (CardioChek PA Analyzer; Polymer Technology Systems, USA). Glucose level was measured by a rapid blood glucose analyzer (BeneCheck BK6–20M Multi-Monitoring System, Suzhou Pu Chun Tang Biotechnology, China). Participants were considered in a fasting state if they had eaten their last meal at least 8 h before their visits. Second, we collected recalled weight at the age of 25 and other individual characteristics through standardized in-person interviews using electronic questionnaires with real-time logical check function. Individual characteristics included age, sex, urbanity, geographic region, education level, household income, smoking status, drinking status, dietary patterns, leisure-time physical activity level, and comorbidities (including diabetes, cardiovascular diseases, chronic obstructive pulmonary diseases [COPD], and cancer). Diabetes was defined if there was a self-report of doctor-diagnosed diabetes or a self-report of use of hypoglycemic drugs. Dietary information was obtained using food frequency questionnaires over the previous year. Three dietary patterns were extracted by factor analysis combined with cluster analysis (16), and the dietary patterns were consistent with those established in previous large-scale studies of Chinese adults with a validated food frequency questionnaire (16, 17). Traditional southern dietary pattern was characterized by a higher intake of rice and a lower intake of wheaten food as staple, as well as a lower intake of protein foods including egg, milk, meat, and seafood. Traditional northern dietary pattern was characterized by a higher intake of wheaten food and a lower intake of rice as staple, and also a lower intake of protein foods. Modern dietary pattern was characterized by high consumption of protein foods and fruit. Leisure-time physical activity was classified according to the 2018 physical activity guideline as (18): (1) sufficient—if participants reported ≥150 min/week of moderate-intensity activity or ≥75 min/week of vigorous-intensity activity; (2) insufficient—if participants reported some physical activity but not enough to meet the definition of sufficient; and (3) inactive—if they reported no moderate-intensity and vigorous-intensity physical activity.

We categorized BMI of young adulthood into three categories according to Chinese obesity guideline (19): underweight (BMI <18.5 kg/m^2^), normal weight (BMI between ≥18.5 and < 24 kg/m^2^), and overweight/obese (BMI ≥24 kg/m^2^). Based on prior studies (20, 21), and also the estimated median (9.2 kg) and 90th percentile (20.9 kg) of weight gain of the study participants, weight gain from young to middle adulthood was categorized as stable weight (weight gain <2.5 kg), moderate weight gain (between ≥2.5 and <10.0 kg), marked weight gain (between ≥10.0 and <20.0 kg), and extreme weight gain (≥20.0 kg). BMI was calculated by dividing weight (kg) by the square of height (m^2^).

The study outcome was metabolic syndrome, defined according to the revised US National Cholesterol Education Program Adult Treatment Panel III (NCEP ATP III) criteria (2004) (22) with modified waist circumference cutoffs based on the latest Chinese obesity guideline (19). Participants diagnosed with metabolic syndrome met 3 or more of the following criteria: (a) high blood pressure—blood pressure ≥130/85 mmHg or use of antihypertensive drugs; (b) hyperglycemia—fasting blood glucose (FBG) level ≥5.6 mmol/l (100 mg/dl) or use of hypoglycemic drugs; (c) high triglycerides (TG)—TG level ≥1.69 mmol/l (150 mg/dl); (d) low high-density lipoprotein cholesterol (HDL-C)—HDL-C level <1.03 mmol/l (40 mg/dl) in men or <1.29 mmol/l (50 mg/dl) in women; and (e) central obesity—waist circumference (WC) ≥90 cm in men and ≥85 cm in women (19, 22).

### Statistical Analysis

Participant characteristics were described across different weight gain categories. Frequencies and percentages were used for categorical variables, and means and standard deviations (SDs) for continuous variables. One-way ANOVA for continuous variable and chi-square test for categorical variable were used for the comparison between different weight gain categories.

Multiple logistic regression models were implemented to assess odds ratios (ORs) of metabolic syndrome associated with per 5-kg weight gain, as well as weight gain categories using stable weight category as a reference. In addition, stratification analyses by BMI categories at the age of 25 were conducted. Covariates used within the logistic regression models (model 1) included age at recruitment (continuous), sex (male and female), geographic region (Eastern, Central and Western), urbanity (rural, urban), education level (primary school or lower, middle school, high school, college or above, unknown), household income (yuan/year: <10,000, 10,000–50,000, >50,000, unknown), smoking status (current smoker, former smoker, never smoker), drinking status (current drinker, non-current drinker), dietary patterns (traditional southern dietary pattern, traditional northern dietary pattern, western dietary pattern), leisure-time physical activity level (sufficient, insufficient, inactive), diabetes (with, without), cardiovascular diseases (with, without), COPD (with, without), and cancer (with, without). Besides, we further adjusted for the current BMI to assess the independent association between weight gain and odds of metabolic syndrome in model 2. Considering that the close relationship between BMI and waist circumference may strongly impact the association between weight change and metabolic syndrome, we applied metabolic disorder instead of metabolic syndrome in the model. The metabolic disorder was defined as having two or more of metabolic syndrome component risk factors excluding waist circumference. In addition, to test the robustness of our results, we conducted sensitivity analysis by applying other waist circumference cutoffs: (1) waist circumference cutoffs (85 cm for men and 80 cm for women) for Chinese population recommended by IDF, NHLBI, AHA, and other institutes (23); and (2) waist circumference cutoffs (90 cm for men and 80 cm for women) for Asian population recommended by AHA and NHLBI (22).

Restricted cubic splines with five knots (5th, 35th, 50th, 65th, and 95th centiles) were applied to model the association of weight gain from young to middle adulthood with ORs of metabolic syndrome and changes of components, adjusting for covariates in model 1 and with or without the current BMI. In addition, stratification analyses by BMI categories at the age of 25 of the association between weight gain from young to middle adulthood and odds of metabolic syndrome applying restricted cubic splines were conducted. The likelihood ratio test was used for the tests for nonlinearity.

To assess the potential selection bias, we compared the participant characteristics among the overall 819,792 participants in this project, 524,604 participants with complete data, and 295,188 participants with missing values. Frequencies and percentages were used for categorical variables, and means and SDs for continuous variables (**Table S1**).

All analyses were conducted using SAS version 9.4 (SAS Institute, Cary, North Carolina, USA). Two tailed tests were used and the P-value < 0.05 was considered to be statistically significant.

## Results

### Participant Characteristics

In total, 437,849 participants were included in the study. The average age was 52.0 years (SD 7.6), and 62.1% were women. Participant characteristics across different weight gain categories were shown in **Table 1**. Overall, from age 25 years to middle age, 12.3% of participants kept stable weight (weight gain <2.5 kg), 40.5% had a moderate amount of weight gain (2.5–10 kg), 35.2% had a marked amount of weight gain (10–20 kg), and 12.0% had an extreme amount of weight gain (≥20 kg). Compared to participants with stable weight or moderate weight gain, those who had marked or extreme weight gain were more likely to be men, current smokers, former smokers and current drinkers, with higher education attainment, and physically active at middle age. They were also more likely to follow traditional northern or modern dietary patterns, live in eastern China and rural areas, earn relatively higher household incomes, and have higher rates of metabolic syndrome, high blood pressure, hyperglycemia, high TG, low HDL-C, central obesity, diabetes, and cardiovascular diseases.

**Table 1:** Participant characteristics at screening by weight gain categories.

	Overall participants included in the present study	Stable weight	Moderate weight gain	Marked weight gain	Extreme weight gain	P-value*
**Participants**	437,849 (100.0)	53,844 ;(12.3)	177,538 (40.5)	154,133 (35.2)	52,334 (12.0)	
**Age, years**	52.0 ± 7.6	52.0 ± 7.9	51.8 ± 7.7	52.1 ± 7.5	52.0 ± 7.4	<0.001
**Women**	271,931 (62.1)	35,916 (66.7)	119,579 (67.4)	91,502 (59.4)	24,934 (47.6)	<0.001
**Leisure time physical activity**						<0.001
Sufficient	126,985 (29.0)	14,004 (26.0)	49,918 (28.1)	47,058 (30.5)	16,005 (30.6)	
Insufficient	9,588 (2.2)	1,048 (1.9)	3,754 (2.1)	3,531 (2.3)	1,255 (2.4)	
Inactive	301,276 (68.8)	38,792 (72.0)	123,866 (69.8)	103,544 (67.2)	35,074 (67.0)	
**Dietary patterns**						<0.001
Modern	38,644 (8.8)	4,801 (8.9)	14,834 (8.4)	13,523 (8.8)	5,486 (10.5)	
Traditional southern	223,802 (51.1)	29,296 (54.4)	94,051 (53.0)	76,412 (49.6)	24,043 (45.9)	
Traditional Northern	175,403 (40.1)	19,747 (36.7)	68,653 (38.7)	64,198 (41.7)	22,805 (43.6)	
**Smoking status**						<0.001
Current smoker	89,254 (20.4)	10,445 (19.4)	32,100 (18.1)	32,809 (21.3)	13,900 (26.6)	
Former smoker	19,566 (4.5)	1,757 (3.3)	6,555 (3.7)	7,830 (5.1)	3,424 (6.5)	
Never smoker	329,029 (75.1)	41,642 (77.3)	138,883 (78.2)	113,494 (73.6)	35,010 (66.9)	
**Current drinker**	4,7514 (10.9)	5,195 (9.6)	16,587 (9.3)	17,743 (11.5)	7,989 (15.3)	<0.001
**Geographic regions**						<0.001
Eastern	165,499 (37.8)	18,324 (34.0)	64,571 (36.4)	60,336 (39.1)	22,268 (42.5)	
Central	136,540 (31.2)	17,190 (31.9)	56,878 (32.0)	47,455 (30.8)	15,017 (28.7)	
Western	135,810 (31.0)	18,330 (34.0)	56,089 (31.6)	46,342 (30.1)	15,049 (28.8)	
**Urbanity**						<0.001
Urban	261,588 (59.7)	33,966 (63.1)	107,125 (60.3)	90,246 (58.6)	30,251 (57.8)	
Rural	17,6261 (40.3)	19,878 (36.9)	70,413 (39.7)	63,887 (41.4)	22,083 (42.2)	
**Education level**						<0.001
Primary school or lower	151,914 (34.7)	21,295 (39.5)	64,389 (36.3)	50,449 (32.7)	15,781 (30.2)	
Middle school	163,690 (37.4)	19,344 (35.9)	65,394 (36.8)	58,516 (38.0)	20,436 (39.0)	
High school	76,222 (17.4)	8,328 (15.5)	29,566 (16.7)	28,172 (18.3)	10,156 (19.4)	
College or above	42,785 (9.8)	4,502 (8.4)	16,918 (9.5)	15,841 (10.3)	5,524 (10.6)	
unknown	3,238 (0.7)	375 (0.7)	1,271 (0.7)	1,155 (0.7)	437 (0.8)	
**Household income (¥/year)**						<0.001
<10,000	63,266 (14.4)	8,506 (15.8)	25,876 (14.6)	21,687 (14.1)	7,197 (13.8)	
10,000–50,000	252,720 (57.7)	31,168 (57.9)	102,690 (57.8)	88,468 (57.4)	30,394 (58.1)	
>50,000	92,054 (21.0)	10,550 (19.6)	36,830 (20.7)	33,433 (21.7)	11,241 (21.5)	
unknown	29,809 (6.8)	3,620 (6.7)	12,142 (6.8)	10,545 (6.8)	3,502 (6.7)	
**Metabolic disorders**						
Metabolic syndrome	197,493 (45.1)	13,360 (24.8)	59,969 (33.8)	83,871 (54.4)	40,293 (77.0)	<0.001
High blood pressure	277,149 (63.3)	28,570 (53.1)	102,339 (57.6)	104,574 (67.8)	41,666 (79.6)	<0.001
Hyperglycemia	273,698 (62.5)	30,522 (56.7)	104,784 (59.0)	100,767 (65.4)	37,625 (71.9)	<0.001
High TG	157,667 (36.0)	13,731 (25.5)	55,112 (31.0)	62,809 (40.7)	26,015 (49.7)	<0.001
Low HDL-C	136,927 (31.3)	12,283 (22.8)	49,262 (27.7)	53,811 (34.9)	21,571 (41.2)	<0.001
Central obesity	184,884 (42.2)	8,814 (16.4)	47,303 (26.6)	83,530 (54.2)	45,237 (86.4)	<0.001
**Comorbidities**						
Diabetes	29,172 (6.7)	2,910 (5.4)	10,054 (5.7)	10,921 (7.1)	5,287 (10.1)	<0.001
Cardiovascular diseases	13,673 (3.1)	1,491 (2.8)	4,712 (2.7)	5,237 (3.4)	2,233 (4.3)	<0.001
COPD	615 (0.1)	73 (0.1)	243 (0.1)	221 (0.1)	78 (0.1)	0.8911
Cancer	619 (0.1)	79 (0.1)	252 (0.1)	220 (0.1)	68 (0.1)	0.8922

Data are the number of participants (percent) or means ± standard deviation. TG, triglycerides; HDL-C, high density lipoprotein cholesterol; COPD, chronic obstructive pulmonary diseases. *One-way ANOVA for means and chi-square test for proportion.

**Figure 1:**
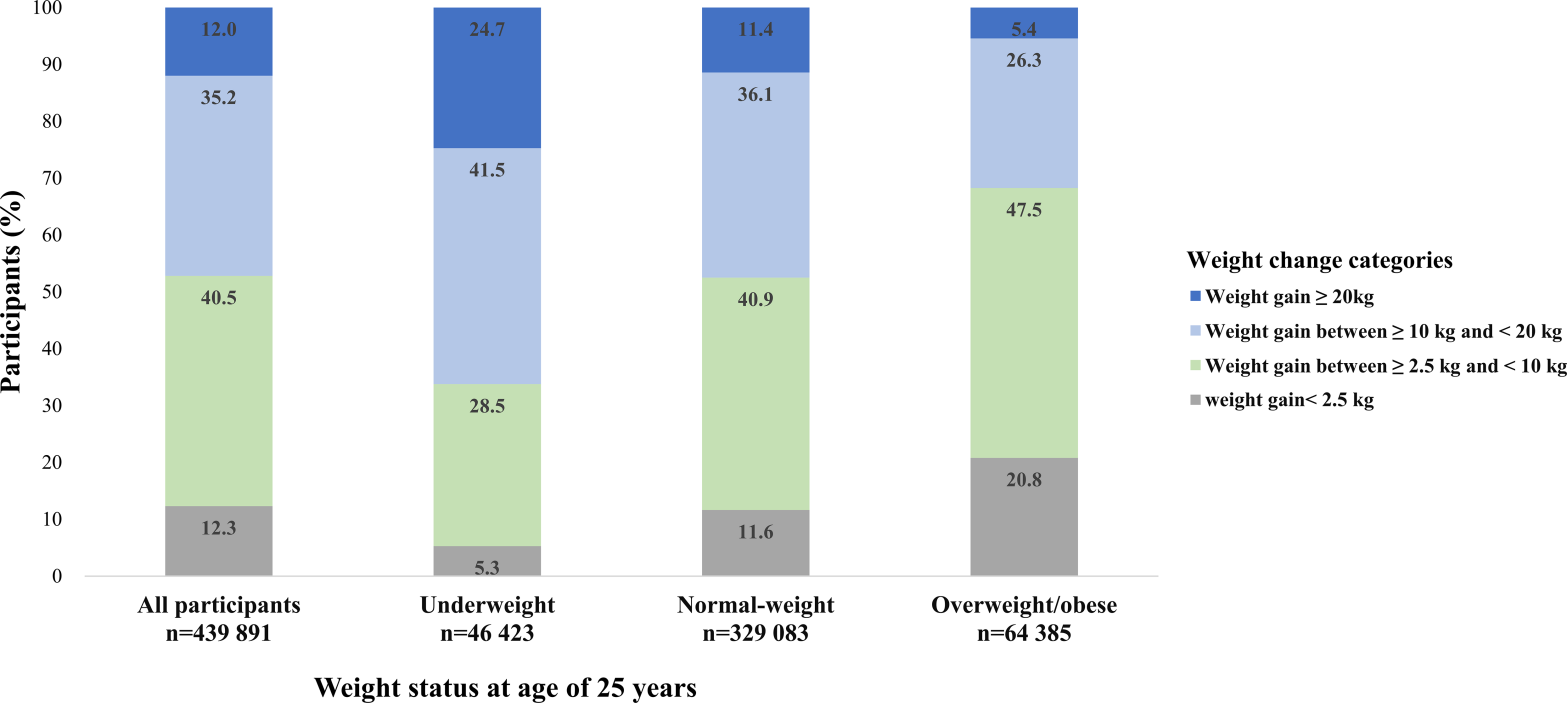
Distribution of weight change categories by BMI categories at young adulthood.

**Figure 2:**
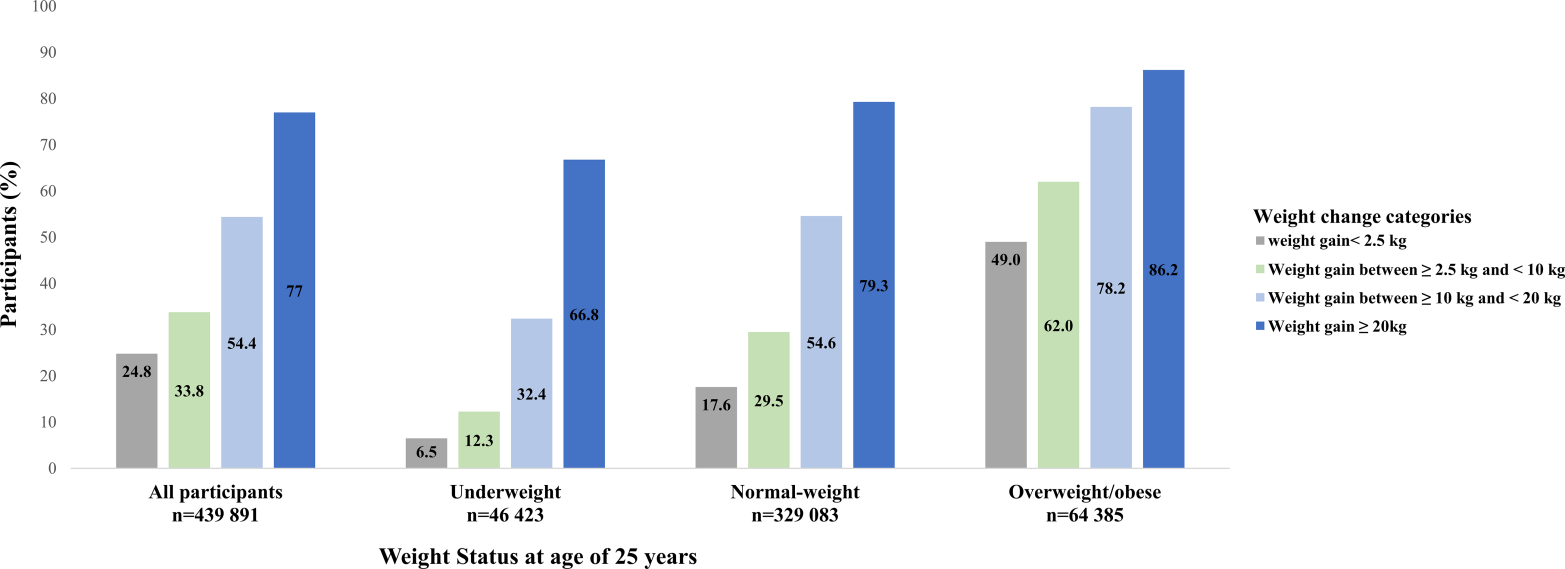
The proportion of participants with metabolic syndrome by BMI categories at age 25 years and weight gain categories from young to middle adulthood.

Participants who were underweight or normal-weight at young adulthood gained more weight than those who were overweight/obese (**Figure 1**). The proportion of participants with metabolic syndrome by BMI categories at age 25 years and weight gain categories was shown in **Figure 2**. Generally, participants who gained more weight or with higher BMI at age 25 years had higher rates of metabolic syndrome.

### Association Between Weight Gain From Young to Middle adulthood and Metabolic Syndrome

The association between weight gain and odds of metabolic syndrome is shown in **Table 2**. In model 1 adjusting for individual risk factors, participants who gained more weight had higher ORs of metabolic syndrome of 1.6 (95% confidence interval [CI]: 1.57–1.64), 3.85 (3.77–3.94), and 11.05 (10.73–11.38) across moderate, marked, and extreme amount of weight gain compared with stable weight, respectively. When stratified by BMI categories at young adulthood, the positive monotonic association was observed in all subgroups. The ORs of metabolic syndrome were 2.01 (1.98–2.05), 1.93 (1.92–1.94) and 1.67 (1.64–1.7) per 5-kg weight gain across underweight, normal-weight and overweight/obese subgroups, respectively. After further adjusting for current BMI, the positive monotonic associations of weight gain with odds of metabolic syndrome were attenuated but remained significant among underweight and normal-weight participants at young adulthood, with ORs of metabolic syndrome of 1.49 (1.41–1.57) and 1.23 (1.22–1.25) per 5-kg weight gain, respectively. While for participants with overweight/obesity at young adulthood, extreme weight gain was not independently associated with metabolic syndrome (OR: 1.03, 95% CI: 0.89–1.18). Similar results were found when we assessed the association between weight gain and metabolic disorder defined as having two or more of metabolic syndrome component risk factors excluding waist circumference (**Table 3**). In addition, similar results were observed when applying waist circumference cutoffs of 85/80 cm (85 cm for men and 80 cm for women) and 90/80 cm (90 cm for men and 80 cm for women) (**Tables S2**, **S3**).

**Table 2:** Odds ratios of metabolic syndrome associated with weight gain from young to middle adulthood by overall and BMI categories at young adulthood.

	Per 5-kg weight gain	Moderate weight gain*	Marked weight gain*	Extreme weight gain*
**All participants**				
Model 1^†^	1.69 (1.68–1.7)	1.6 (1.57–1.64)	3.85 (3.77–3.94)	11.05 (10.73–11.38)
Model 2^‡^	1.17 (1.17–1.18)	1.14 (1.12–1.17)	1.54 (1.51–1.58)	1.92 (1.86–1.99)
**Underweight**				
Model 1^†^	2.01 (1.98–2.05)	2.05 (1.72–2.44)	6.82 (5.76–8.06)	30.5 (25.73–36.16)
Model 2^‡^	1.49 (1.41–1.57)	1.11 (0.93–1.32)	1.53 (1.27–1.85)	2.23 (1.79–2.77)
**Normal–weight**				
Model 1^†^	1.93 (1.92–1.94)	2.01 (1.95–2.07)	5.93 (5.76–6.11)	19.48 (18.76–20.22)
Model 2^‡^	1.23 (1.22–1.25)	1.2 (1.17–1.24)	1.69 (1.64–1.76)	2.05 (1.95–2.15)
**Overweight/obese**				
Model 1^†^	1.67 (1.64–1.7)	1.78 (1.7–1.85)	4.08 (3.88–4.3)	7.22 (6.5–8.01)
Model 2^‡^	1.11 (1.08–1.14)	1.21 (1.16–1.27)	1.43 (1.33–1.53)	1.03 (0.89–1.18)

Data are odds ratios (95% confidence interval).

*Stable weight was used as reference (weight gain <2.5 kg).

^†^Model 1 adjusted for age, sex, geographic region, urbanity, education level, income, smoking status, drinking status, dietary patterns, leisure time physical activity level, and comorbidities.

^‡^Model 2 additionally adjusted for current BMI.

**Table 3:** Odds ratios of metabolic disorder* associated with weight gain from young to middle adulthood by overall and BMI categories at young adulthood.

	Per 5-kg weight gain	Moderate weight gain^†^	Marked weight gain^†^	Extreme weight gain^†^
**All participants**				
Model 1^‡^	1.76 (1.75–1.77)	1.53 (1.5–1.56)	3.74 (3.65–3.82)	14.09 (13.53–14.68)
Model 2^§^	1.2 (1.19–1.21)	1.07 (1.04–1.09)	1.42 (1.38–1.46)	2.25 (2.15–2.36)
**Underweight**				
Model 1^‡^	1.89 (1.86–1.92)	1.57 (1.42–1.73)	4.04 (3.67–4.44)	18.41 (16.52–20.52)
Model 2^§^	1.44 (1.37–1.52)	0.9 (0.81–1)	1.06 (0.94–1.21)	1.75 (1.46–2.09)
**Normal-weight**				
Model 1^‡^	1.98 (1.97–2)	1.75 (1.7–1.79)	5.01 (4.89–5.15)	23.73 (22.5–25.02)
Model 2^§^	1.28 (1.26–1.29)	1.07 (1.04–1.09)	1.47 (1.43–1.52)	2.57 (2.41–2.75)
**Overweight/obese**				
Model 1^‡^	2.09 (2.03–2.16)	1.9 (1.8–2)	6.22 (5.72–6.75)	18.57 (14.29–24.12)
Model 2^§^	1.16 (1.1–1.21)	1.08 (1.02–1.15)	1.36 (1.21–1.53)	1.14 (0.84–1.53)

Data are odds ratios (95% confidence interval).

*Metabolic disorder was defined by two or more of metabolic syndrome component risk factors excluding waist circumference.

^†^Stable weight was used as reference (weight gain <2.5 kg).

^‡^Model 1 adjusted for age, sex, geographic region, urbanity, education level, income, smoking status, drinking status, dietary patterns, leisure time physical activity level, and comorbidities.

^§^Model 2 additionally adjusted for current BMI.

**Figure 3:**
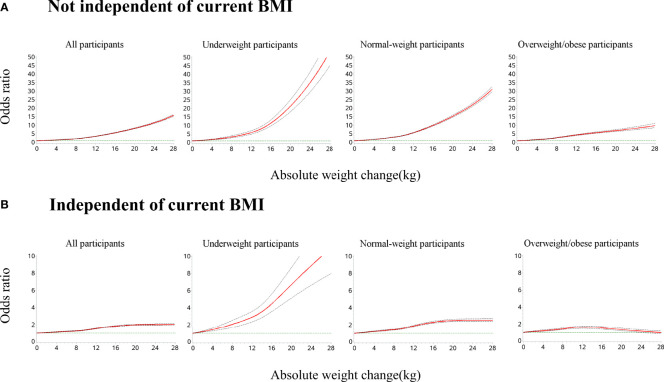
Dose–response association of weight gain from young to middle adulthood with risk of metabolic syndrome across BMI categories at young adulthood. Restricted cubic splines were used with five knots (5th, 35th, 50th, 65th, and 95th centiles). Reference point is 0 kg for weight gain. Odds ratios were indicated by solid lines and 95% confidence intervals by dashed lines. **(A)** All models were adjusted for age, sex, geographic region, urbanity, education level, income, smoking status, drinking status, dietary patterns, leisure time physical activity level, and comorbidities. **(B)** All models were adjusted for confounders in **(A)** and current BMI. P for non-linearity <0.001 in all models.

In the restricted cubic splines of weight gain and odds of metabolic syndrome (**Figure 3**), for underweight and normal-weight participants at young adulthood, the odds of metabolic syndrome increased with a higher amount of weight gain and the ascending slope was steeper at a higher amount of weight gain. For participants with overweight/obesity at young adulthood, the curve was nearly linear. The association was stronger among underweight or normal-weight participants than participants with overweight/obesity at young adulthood. After further adjusting for the current BMI, for underweight and normal-weight participants, the odds of metabolic syndrome increased with larger weight gains. For participants with overweight/obesity, an inverted U-shaped association was observed: the odds of metabolic syndrome increased with increasing weight gain until around 12 kg and then decreased (P for non-linearity <0.001).

We further assessed the association of weight gain from young to middle adulthood with the change in metabolic syndrome components (**Figure S2**). For all subgroups of participants with different BMI categories at young adulthood, weight gain was associated with adverse changes in metabolic syndrome components. All of the associations were slightly stronger among participants who were underweight or normal-weight than those with overweight/obesity at young adulthood, only except for WC.

The demographic, socioeconomic, and clinical characteristics were comparable among the overall 819,792 participants in this project, 524,604 participants with complete data, and 295,188 participants with missing values. The participants with complete data were more likely to be physically active and follow traditional northern dietary pattern.

## Discussion

In this large, national population-based study, we found that larger weight gains from young to middle adulthood were associated with higher odds of metabolic syndrome at middle adulthood across all BMI categories at young adulthood, especially among underweight and normal-weight participants. After further accounting for the current BMI, larger weight gains were still associated with higher odds of metabolic syndrome, with the exception of overweight/obesity people, in whom an inverted U-shaped association was observed. Weight maintenance could be effective to mitigate metabolic syndrome burden across all BMI categories at young adulthood.

To our knowledge, we found no other studies testing the dose–response association between weight gain from young to middle adulthood and metabolic syndrome risk across different BMI categories at young adulthood. We found that any amount of weight gain was shown to be associated with higher odds of metabolic syndrome across different BMI categories at young adulthood. Prior studies also found the positive association across different BMI categories at young adulthood, while they used dichotomous variables of weight gain and weight stable, not considering the amount of weight gain (7, 8, 12). Of note, we observed that for normal-weight individuals at young adulthood, this relationship became particularly stronger after the weight gain was over around 10 kg. Given the difficulty of maintaining weight during adulthood, keeping weight gain less than 10 kg may be a suboptimal goal to keep metabolic health for normal-weight individuals at young adulthood.

Prior study showed that underweight is related to lower risk of metabolic syndrome than normal weight and obese (24). Our study further found that weight change, from underweight at young adulthood to normal weight at middle adulthood, was associated with higher odds of metabolic syndrome compared with keeping underweight. However, for underweight people, the effects of maitaining underweight on the overall risk, namely, cardiac and non-cardiac risk, are unclear. A U-shaped association was reported between BMI and mortality in middle aged or older people (25). Moreover, in several recent studies, BMI in early adulthood was shown to be linearly associated with mortality in later life (21). Further exploration is needed for people who were underweight at young adulthood to determine the optimal weight management strategy to achieve best health outcomes.

In our study, the weight gain from young to middle adulthood was more strongly associated with odds of metabolic syndrome among people who were underweight or normal-weight than those with overweight/obesity at young adulthood. Similar findings were observed in a study by Suzuki et al., showing that weight gain at age 20 years in non-obese individuals was related to more items of adverse metabolic risk factors than in obese individuals (8). In addition, in a Dutch cohort study, the effect of a 5-year weight change on metabolic syndrome risk was slightly weaker in persons with a higher baseline weight (26). BMI categories at young adulthood may be related with the differences in genetic deposition (14), health behaviors (27), socioeconomic status (28), and body composition; these factors may further influence the effects of weight gained. Further studies are needed to explore the underlying mechanisms regarding how BMI categories at young adulthood affect the association between weight gain and metabolic risk.

Our findings indicated that weight gain from young to middle adulthood was associated with increased odds of metabolic syndrome independent of the current BMI, except for people with overweight/obesity at young adulthood. This suggests that adult weight gain may confer additional metabolic syndrome risk besides the risk mediated by a larger attained BMI. Similar findings were observed in the US National Health and Nutrition Examination Survey, in which BMI categories at young adulthood were not considered (10). The possible mechanism might be that weight gain from young to middle adulthood is mainly attributable to adipose tissue growth (29, 30) and more accumulation of visceral adipose tissue (31, 32), a well-established risk factor for metabolic disorders (33). Hence, the weight gain during adulthood may contribute to the heterogeneity in metabolic status among individuals with similar current BMI. While for people with overweight/obesity, duration of obesity may lead to compensatory adaptations to maintain insulin sensitivity (34). This may explain the results observed in our study that larger weight gains in people with overweight/obesity were not associated with increased odds of metabolic syndrome independent of the current BMI.

Several limitations should be considered when interpreting the findings in the current study. Firstly, we used self-reported body weight at the age of 25, hence the results might be influenced by recall bias. A previous study showed that there might be the phenomenon of “regression to the mean” when participants recalled their body weight (heavy persons tend to underestimate their weight and thin persons tend to overestimate their weight) (35, 36). Therefore, the relationship between weight gain and odds of metabolic syndrome might be underestimated in overweight/obese people, while overestimated in underweight people. However, prior validation study showed good validity of recalled early life bodyweight which could be used in life-course epidemiological studies (37). In addition, many large-scale studies also applied the self-reported and recalled body weight (20, 21). Secondly, we could not obtain other measures of body weight between age 25 years and middle age, therefore we could not determine weight cycling during this period. Thirdly, we applied the variable of weight gain assessed by current body weight minus historical body weight, reflecting changes in body weight over time. Nevertheles, the cross-sectional design should still be cautious about reverse causality. Fourthly, several unmeasured confounders may exist, such as lifestyle changes from young to middle adulthood and activities or attempts related to weight loss, which may lead to potential bias. Fifthly, our study only included Chinese individuals, which might limit the potential generalizability of the results to other populations. Sixthly, potential selection bias might exist because participants included in our study were more likely to be physically active and follow traditional northern dietary pattern compared with the overall participants in the project. Hence, our results might be more applicable to the population who are more physically active or more likely to follow traditional northern dietary pattern.

## Conclusions

This national population-based study indicates that any amount of weight gain from young to middle adulthood is associated with increased odds of metabolic syndrome across all subgroups with different BMI categories, particularly underweight and normal-weight at young adulthood, even after accounting for the current BMI. Our findings provided valuable information for strategy-making of body weight management to mitigate metabolic syndrome risk.

## Data Availability Statement

The original contributions presented in the study are included in the article/**Supplementary Material**, further inquiries can be directed to the corresponding authors.

## Ethics Statement

The studies involving human participants were reviewed and approved by the central ethics committee at Fuwai Hospital. The patients/participants provided their written informed consent to participate in this study.

## Author Contributions

XW, JML, and XZhe conceived and designed the study. XW wrote the draft of the article. XW and YG did the statistical analysis. JML and XZhe take responsibility for the study supervision. All authors contributed to the article and approved the submitted version

## Funding

This work was supported by the National Key Research and Development Program (2020YFC2004703) from the Ministry of Science and Technology of China, the China Academy of Chinese Medical Sciences Innovation Fund for Medical Science (2021-I2M-1-009).

## Conflict of Interest

The authors declare that the research was conducted in the absence of any commercial or financial relationships that could be construed as a potential conflict of interest.

## Publisher’s Note

All claims expressed in this article are solely those of the authors and do not necessarily represent those of their affiliated organizations, or those of the publisher, the editors and the reviewers. Any product that may be evaluated in this article, or claim that may be made by its manufacturer, is not guaranteed or endorsed by the publisher.
