# Supplementary material for: Association Between Weight Gain From Young to Middle Adulthood and Metabolic Syndrome Across Different BMI Categories at Young Adulthood
Source: Front Endocrinol (Lausanne). 2022 Feb 15;12:812104. doi: 10.3389/fendo.2021.812104 (PMC8886729; doi:10.3389/fendo.2021.812104)
Supplement: Supplementary file 1 [file DataSheet_1.docx]

Supplementary Material

**Figure S1 Flowchart of study population**

**
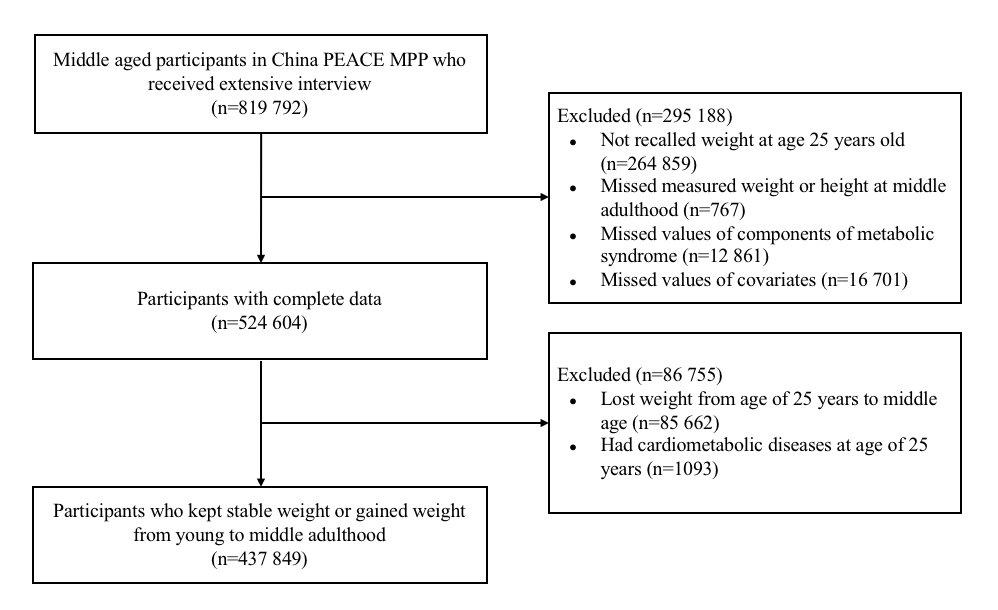
**

**Figure S2 Dose-response association of weight gain from young to middle adulthood with change in component risk factors across BMI categories at young adulthood.**

**
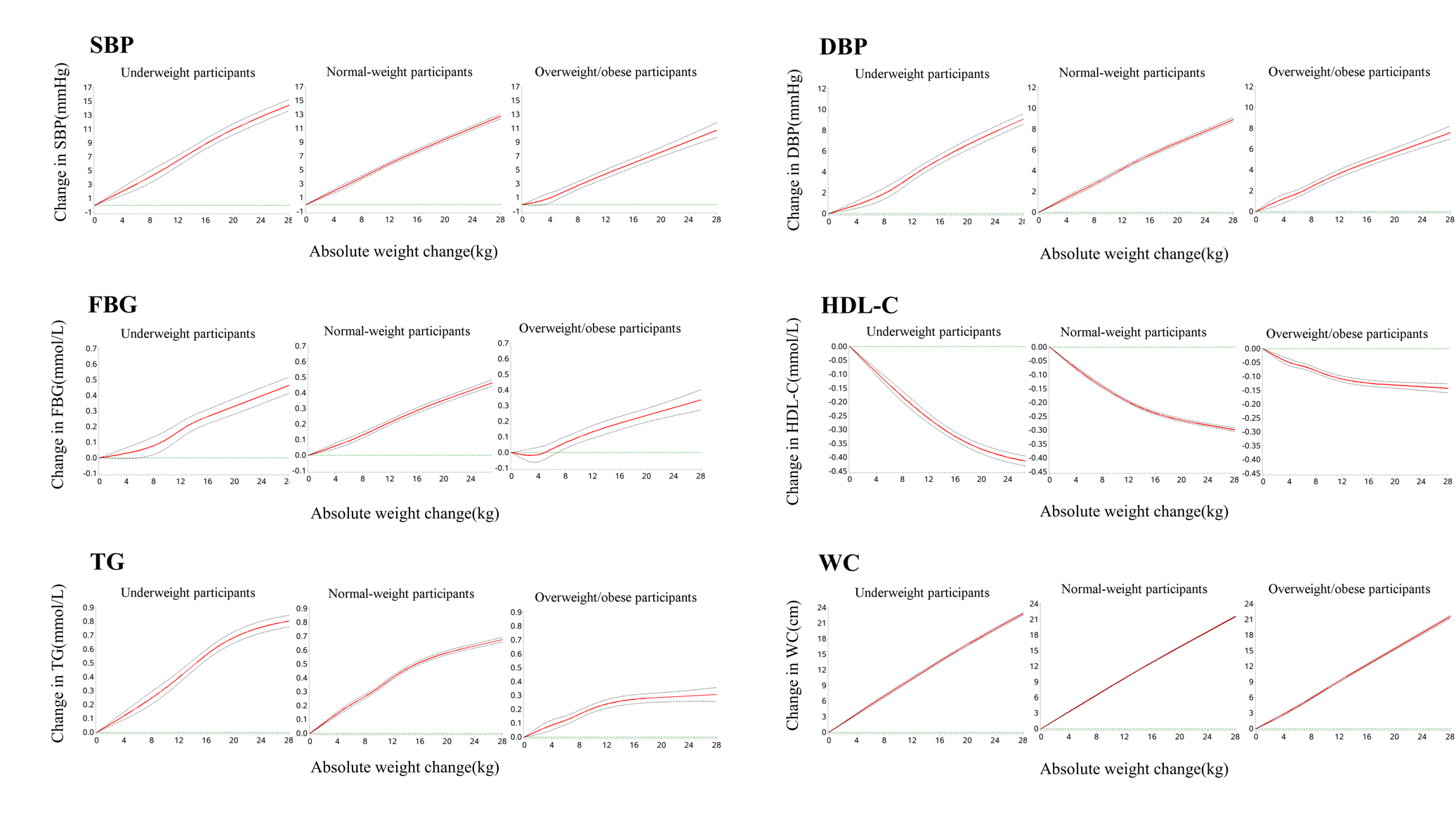
**

Participants who took antihypertensive drugs were excluded for analysis of SBP and DBP, and participants who took hypoglycemic drugs were excluded for analysis of FBP. Restricted cubic splines were used with five knots (5th, 35th, 50th, 65th, 95th centiles). Reference point is 0 kg for weight gain. The estimated change in component risk factors was indicated by solid lines and 95% confidence intervals by dashed lines. All models were adjusted for age, sex, geographic region, urbanity, education level, income, health insurance status, smoking status, drinking status, dietary patterns, and leisure time physical activity level. Abbreviations: SBP=systolic blood pressure, DBP=diastolic blood pressure, FBG= fasting blood glucose, HDL-C= high-density lipoprotein cholesterol, TG= triglyceride, WC=waist circumference.

**Table S1 Participant characteristics at screening among the overall participants, participants with complete data, and participants with missing values in the project.**

|  | **Participants with missing values** | **Participants with complete data** | **Overall participants in the project** |
| --- | --- | --- | --- |
| **Participants** | 295188 (36.0) | 524604 (64.0) | 819792 (100.0) |
| **Age, years** | 51.9 ± 7.8 | 52.2 ± 7.6 | 52.1 ± 7.7 |
| **Women** | 175566(59.5) | 327836(62.5) | 503402(61.4) |
| **Leisure time physical activity** |  |  |  |
| Sufficient | 39841(15.8) | 149965(28.6) | 189806(24.5) |
| Insufficient | 2770(1.1) | 11168(2.1) | 13938(1.8) |
| Inactive | 208990(83.1) | 363471(69.3) | 572461(73.8) |
| **Dietary patterns** |  |  |  |
| Modern | 50963(17.3) | 46106(8.8) | 97069(11.8) |
| Traditional Southern | 148545(50.3) | 270069(51.5) | 418614(51.1) |
| Traditional Northern | 77627(26.3) | 208429(39.7) | 286056(34.9) |
| **Smoking status** |  |  |  |
| Current smoker | 57245(19.5) | 108821(20.7) | 166066(20.3) |
| Former smoker | 8891(3.0) | 22947(4.4) | 31838(3.9) |
| Never smoker | 227637(77.5) | 392836(74.9) | 620473(75.8) |
| **Current drinker** | 25477(8.8) | 56609(10.8) | 82086(10.1) |
| **Geographic regions** |  |  |  |
| Eastern | 104296(35.3) | 193350(36.9) | 297646(36.3) |
| Central | 77529(26.3) | 164082(31.3) | 241611(29.5) |
| Western | 113363(38.4) | 167172(31.9) | 280535(34.2) |
| **Urbanity** |  |  |  |
| Urban | 179604(60.8) | 317760(60.6) | 497364(60.7) |
| Rural | 115584(39.2) | 206844(39.4) | 322428(39.3) |
| **Education level** |  |  |  |
| Primary school or lower | 120877(41.0) | 189815(36.2) | 310692(37.9) |
| Middle school | 96942(32.8) | 193465(36.9) | 290407(35.4) |
| High school | 47766(16.2) | 89025(17.0) | 136791(16.7) |
| College or above | 24189(8.2) | 48483(9.2) | 72672(8.9) |
| unknown | 5404(1.8) | 3816(0.7) | 9220(1.1) |
| **Household income (¥/year)** |  |  |  |
| <10 000 | 49965(16.9) | 78963(15.1) | 128928(15.7) |
| 10 000-50 000 | 159448(54.0) | 302624(57.7) | 462072(56.4) |
| >50 000 | 48544(16.4) | 107027(20.4) | 155571(19.0) |
| unknown | 37218(12.6) | 35990(6.9) | 73208(8.9) |
| **Metabolic disorders** |  |  |  |
| Metabolic syndrome | 103534(37.8) | 217878(41.5) | 321412(40.2) |
| High blood pressure | 167592(56.8) | 325189(62.0) | 492781(60.1) |
| Hyperglycemia | 171199(60.5) | 324516(61.9) | 495715(61.4) |
| High TG | 92206(31.8) | 177843(33.9) | 270049(33.2) |
| Low HDL-C | 86124(29.6) | 154868(29.5) | 240992(29.5) |
| Central obesity | 104140(35.3) | 197487(37.6) | 301627(36.8) |
| **Comorbidities** |  |  |  |
| Diabetes | 16554(5.6) | 37248(7.1) | 53802(6.6) |
| Cardiovascular diseases | 5479(1.9) | 17004(3.2) | 22483(2.7) |
| COPD | 402(0.1) | 882(0.2) | 1284(0.2) |
| Cancer | 347(0.1) | 786(0.1) | 1133(0.1) |

Data are the number of participants (percent) or means ± standard deviation. Abbreviations: TG=triglycerides, HDL-C=high density lipoprotein cholesterol, COPD= chronic obstructive pulmonary diseases.

**Table S2 Odds ratios of metabolic syndrome according to NCEP ATP III criteria with modified waist circumference of 85/80 cm associated with weight gain from young to middle adulthood by overall and BMI categories at young adulthood.**

|  | **Per 5–kg weight gain** | **Moderate weight gain*** | **Marked weight gain*** | **Extreme weight gain*** |
| --- | --- | --- | --- | --- |
| **All participants** |  |  |  |  |
| Model 1† | 1.65(1.64-1.66) | 1.67(1.64-1.71) | 3.98(3.9-4.07) | 9.46(9.19-9.74) |
| Model 2‡ | 1.15(1.14-1.16) | 1.21(1.18-1.24) | 1.64(1.6-1.68) | 1.7(1.64-1.76) |
| **Underweight** |  |  |  |  |
| Model 1† | 2.07(2.04-2.11) | 2.25(1.91-2.65) | 9.26(7.91-10.84) | 37.61(32.02-44.19) |
| Model 2‡ | 1.44(1.37-1.52)  1.44(1.37-1.52) | 1.22(1.04-1.44) | 2.14(1.79-2.55) | 2.86(2.32-3.53) |
| **Normal–weight** |  |  |  |  |
| Model 1† | 1.86(1.85-1.87) | 2.18(2.12-2.24) | 6.24(6.07-6.41) | 15.68(15.12-16.26) |
| Model 2‡ | 1.18(1.17-1.19) | 1.33(1.29-1.36) | 1.83(1.77-1.9) | 1.69(1.61-1.77) |
| **Overweight/obese** |  |  |  |  |
| Model 1† | 1.48(1.46-1.51) | 1.63(1.56-1.7) | 2.99(2.84-3.16) | 4.69(4.22-5.21) |
| Model 2‡ | 1.07(1.04-1.1) | 1.2(1.15-1.26) | 1.31(1.22-1.41) | 1.02(0.89-1.17) |

Data are odds ratios (95% confidence interval). *Stable weight was used as reference (weight gain < 2.5 kg). †Model 1 adjusted for age, sex, geographic region, urbanity, education level, income, smoking status, drinking status, dietary patterns, leisure time physical activity level, and comorbidities. ‡Model 2 additionally adjusted for current BMI. Metabolic syndrome was defined as meeting 3 or more of the following criteria: (a) high blood pressure–blood pressure ≥ 130/85 mmHg or use of antihypertensive drugs, (b) hyperglycemia–fasting blood glucose (FBG) level ≥ 5.6 mmol/l (100 mg/dl) or use of hypoglycemic drugs, (c) high triglycerides (TG)–TG level ≥ 1.69 mmol/l (150 mg/dl), (d) low high-density lipoprotein cholesterol (HDL-C)–HDL-C level < 1.03 mmol/l (40 mg/dl) in men or < 1.29 mmol/l (50 mg/dl) in women, and (e) central obesity–waist circumference (WC) ≥ 85 cm in men and ≥ 80 cm in women.

**Table S3 Odds ratios of metabolic syndrome according to NCEP ATP III criteria with modified waist circumference of 90/80 cm associated with weight gain from young to middle adulthood by overall and BMI categories at young adulthood.**

|  | **Per 5–kg weight gain** | **Moderate weight gain*** | **Marked weight gain*** | **Extreme weight gain*** |
| --- | --- | --- | --- | --- |
| **All participants** |  |  |  |  |
| Model 1† | 1.67(1.66-1.68) | 1.63(1.59-1.66) | 3.88(3.79-3.96) | 10.31(10.02-10.62) |
| Model 2‡ | 1.17(1.16-1.17) | 1.17(1.14-1.2) | 1.58(1.54-1.62) | 1.85(1.79-1.92) |
| **Underweight** |  |  |  |  |
| Model 1† | 2.06(2.03-2.1) | 2.28(1.93-2.7) | 8.88(7.56-10.44) | 37(31.36-43.64) |
| Model 2‡ | 1.48(1.41-1.56)  1.44(1.37-1.52) | 1.22(1.03-1.44) | 1.98(1.66-2.37) | 2.66(2.15-3.3) |
| **Normal–weight** |  |  |  |  |
| Model 1† | 1.9(1.88-1.91) | 2.09(2.03-2.15) | 6.02(5.85-6.19) | 17.9(17.26-18.57) |
| Model 2‡ | 1.21(1.2-1.23) | 1.27(1.23-1.3) | 1.75(1.7-1.81) | 1.95(1.85-2.05) |
| **Overweight/obese** |  |  |  |  |
| Model 1† | 1.55(1.53-1.58) | 1.65(1.58-1.72) | 3.34(3.17-3.52) | 5.58(5.02-6.2) |
| Model 2‡ | 1.09(1.06-1.12) | 1.19(1.13-1.24) | 1.37(1.27-1.47) | 1.07(0.93-1.23) |

Data are odds ratios (95% confidence interval). *Stable weight was used as reference (weight gain < 2.5 kg). †Model 1 adjusted for age, sex, geographic region, urbanity, education level, income, smoking status, drinking status, dietary patterns, leisure time physical activity level, and comorbidities. ‡Model 2 additionally adjusted for current BMI. Metabolic syndrome was defined as meeting 3 or more of the following criteria: (a) high blood pressure–blood pressure ≥ 130/85 mmHg or use of antihypertensive drugs, (b) hyperglycemia–fasting blood glucose (FBG) level ≥ 5.6 mmol/l (100 mg/dl) or use of hypoglycemic drugs, (c) high triglycerides (TG)–TG level ≥ 1.69 mmol/l (150 mg/dl), (d) low high-density lipoprotein cholesterol (HDL-C)–HDL-C level < 1.03 mmol/l (40 mg/dl) in men or < 1.29 mmol/l (50 mg/dl) in women, and (e) central obesity–waist circumference (WC) ≥ 90 cm in men and ≥ 80 cm in women.
